# Development of a Mobile App for Self-Care Against COVID-19 Using the Analysis, Design, Development, Implementation, and Evaluation (ADDIE) Model: Methodological Study

**DOI:** 10.2196/39718

**Published:** 2022-09-13

**Authors:** Hamid Reza Saeidnia, Marcin Kozak, Marcel Ausloos, Claudiu Herteliu, Zahra Mohammadzadeh, Ali Ghorbi, Mehrdad Karajizadeh, Mohammad Hassanzadeh

**Affiliations:** 1 Department of Knowledge and Information Science Tarbiat Modares University Tehran Iran; 2 Department of Media, Journalism and Social Communication University of Information Technology and Management in Rzeszow Rzeszow Poland; 3 School of Business University of Leicester Brookfield, Leicester United Kingdom; 4 Department of Statistics and Econometrics Bucharest University of Economic Studies Bucharest Romania; 5 Department of Health Information Management and Technology School of Allied Medical Sciences Kashan University of Medical Sciences Kashan Iran; 6 Department of Knowledge and Information Science University of Tehran Tehran Iran; 7 Trauma Research Center Shahid Rajaee (Emtiaz) Trauma Hospital Shiraz University of Medical Sciences Shiraz, Fars Iran

**Keywords:** self-care, mobile app, ADDIE model, COVID-19, underdeveloped countries

## Abstract

**Background:**

Mobile apps have been shown to play an important role in the management, care, and prevention of infectious diseases. Thus, skills for self-care—one of the most effective ways to prevent illness—can be improved through mobile health apps.

**Objective:**

This study aimed to design, develop, and evaluate an educational mobile-based self-care app in order to help the self-prevention of COVID-19 in underdeveloped countries. We intended the app to be easy to use, quick, and inexpensive.

**Methods:**

In 2020 and 2021, we conducted a methodological study. Using the ADDIE (analysis, design, development, implementation, and evaluation) educational model, we developed a self-care management mobile app. According to the ADDIE model, an effective training and performance support tool is built through the 5 phases that comprise its name. There were 27 participants who conducted 2 evaluations of the mobile app’s usability and impact using the mobile health app usability and self-care inventory scales. The study design included pre- and posttesting.

**Results:**

An Android app called MyShield was developed. The results of pre- and posttests showed that on a scale from 0 to 5, MyShield scored a performance average of 4.17 in the physical health dimension and an average of 3.88 in the mental well-being dimension, thereby showing positive effects on self-care skills. MyShield scored highly on the “interface and satisfaction,” “ease of use,” and “usefulness” components.

**Conclusions:**

MyShield facilitates learning self-care skills at home, even during quarantine, increasing acquisition of information. Given its low development cost and the ADDIE educational design on which it is based, the app can be helpful in underdeveloped countries. Thus, low-income countries—often lacking other tools—can use the app as an effective tool for fighting COVID-19, if it becomes a standard mobile app recommended by the government.

## Introduction

In 2019 and 2020, COVID-19 attracted international attention as a serious threat to public health [1,2]. Thereafter, the virus spread rapidly around the world. On March 11, 2020, the World Health Organization declared the epidemic to be a pandemic [3]. The world then faced a situation that seemed impossible: without war, the global population was in danger; COVID fatalities mounted, and nothing seemed to make people safe. What remained was hope, and small steps everyone could take, such as isolation and good hygiene. This is where simple tools can help, and some examples of such tools are those related to digital technology.

Higher-income and further-developed countries such as Japan, the United States, and South Korea indeed started using digital technology to fight COVID-19, including such methods as contact tracing, the internet of things, big-data analytics, and artificial intelligence [4]. In other, similarly developed countries, such as Canada and Australia, mobile apps (eg, COVID Alert and COVIDSafe) were used to inform citizens about COVID-19 and its prevention [5-7]. These tools appeared to be working well: with a relatively low usage cost to both the governmental and individual user, their effects were quite noticeable.

Nonetheless, most people in lower-income and underdeveloped countries lack access to complex digital technology, as these countries’ levels of information and communications technology infrastructure are much lower than those of more developed countries [8,9]. Several studies have recently indicated that mortality, hospitalization rate, and complications due to COVID-19 in underdeveloped countries have been higher than in other countries [8-10]. It is conventionally agreed upon that due to demographic features, weak health care systems, inequality in the economic and political sectors, corruption, and other sociocultural characteristics, many underdeveloped countries have lower levels of protection against the spread of COVID-19 and its subsequent effects [8,9,11]. In addition to these factors, a lack of social distancing and self-care knowledge contributed to an increased incidence of COVID-19 and its variations, highlighted by a mutation first reported in India commonly known as the Delta variant [12]. All viruses have variants, but the coronavirus’ new mutations make it unpredictable and even more difficult to fight.

For the above reasons, learning self-care is important [13,14]. For lower-income and socially unequal communities, self-care is considered to be the main way of responding to the disease’s spread and severity, mainly because of self-care’s low cost [15]. In self-care, individuals use their own knowledge and abilities to maintain and improve their health [16]. It is a challenging skill that requires time and energy, because its implementation depends on internal (ie, cognitive, physical, emotional, and behavioral) and external (ie, environmental, political, and societal) factors [14]. Meanwhile, self-care has become a new strategy for managing and preventing chronic and infectious diseases, leading to increased energy, more positive emotions, reduced stress, improved health and well-being, and increased self-confidence [17,18]. Self-care is where mobile apps enter the scene, with their capabilities to help people learn self-care tasks.

COVID-19–related smartphone apps offer useful capabilities and functionalities, such as tracking, follow-up, and prevention [18-20]. Furthermore, the growing popularity of mobile software and its relatively low cost have led to the emergence of mobile self-care systems [21]. Not only are such systems relatively cheap and easy to develop, but they also help to manage disease prevention. It is no wonder that in recent years, mobile software has been applied in various medical and health fields [18-21]. In light of these advancements, current digital technologies are highly productive despite their low cost, and they can greatly support health management in lower-income and underdeveloped countries.

While a basic mobile app can be built solely by a person or persons with sufficient development skills, such an app is unlikely to become a useful, popular app; it has been built using only the knowledge and skills of the developer or developers, without input or feedback from specialized experts. Knowing this, we decided to design our app with deeper knowledge in the areas of mobile app design, development, and COVID-19 health information. This involved conducting a study in which we would ask various experts—health practitioners and mobile technology professionals—for their help; in this way, the resulting app would be designed using much deeper and broader knowledge than a single author or team could provide.

We also decided to use the ADDIE (analysis, design, development, implementation, and evaluation) education model, which is used to build educational systems. While the app we wanted to create was not an educational system per se, it did aim to teach, and we wanted it to teach effectively. We observed a strong correlation between our concept of a mobile app and educational systems created using the ADDIE model. Why did we decide to use this model to create a mobile app? We believed that an app that was designed using an educational model had a greater chance to teach its users effectively than an app designed without an educational methodology.

Therefore, in this study, we aimed to design, develop, and implement a standalone mobile app for training self-care skills based on the ADDIE educational model in order to improve self-care and prevent COVID-19, particularly in underdeveloped countries. We evaluated various facets of the app, all directly or indirectly related to its main goal: to help its users enhance their self-care skills in the context of COVID-19.

## Methods

### Overview

**Figure 1:**
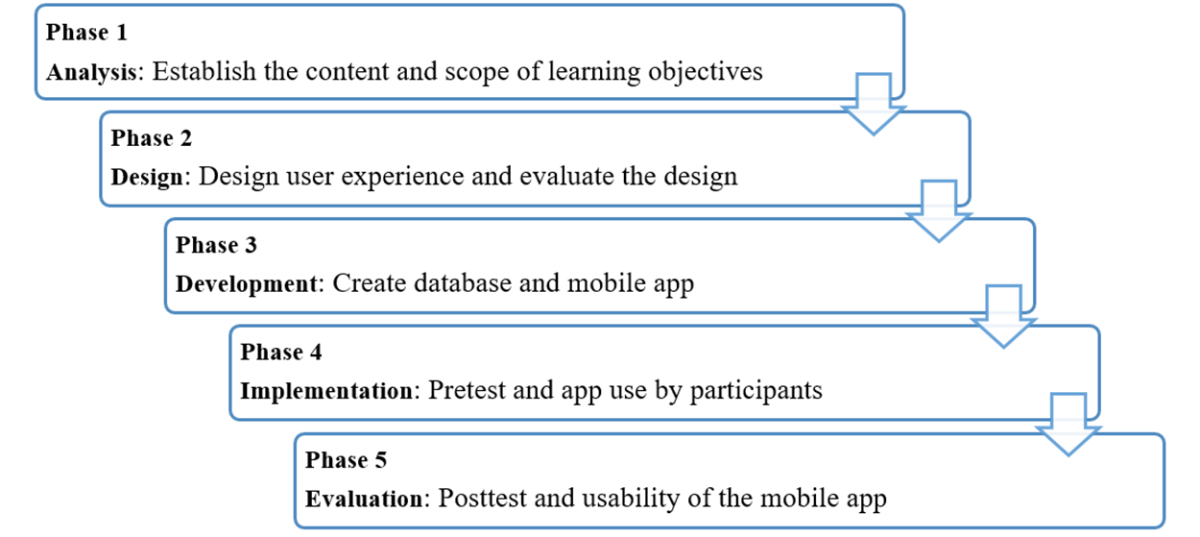
Educational app development (analysis, design, development, implementation, and evaluation) model phases.

The present methodological study used the ADDIE educational design model to develop an educational mobile app for self-care with the purpose of supporting self-prevention of COVID-19 [22]. ADDIE gives education professionals and designers the ability to lay the groundwork for principled and effective training [23]. This model can be utilized for both traditional and electronic learning [24]. ADDIE consists of the 5 phases the acronym was built upon: analysis, design, development, implementation, and evaluation (Figure 1) [23].

### Analysis Phase

During the analysis phase, we sought to determine the appropriate practical contents of the mobile app and categorize them. For this purpose, from October 1 to 15, 2020, we interviewed 3 infectious disease experts and 2 nursing specialists. Because of COVID-19 conditions, the interviews were remote and used the Zoom meeting software. We used qualitative methods to analyze and categorize the resulting content requirements for a self-care mobile app [18,25]. In order to make the app pleasant to use, we used interesting content presented in various formats (eg, written text, images, infographics, and videos).

### Design Phase

In the design phase, we focused on optimizing the user experience of the designed mobile app and its feature list; we did so through online workshops with a group of experts in the fields of health information technology, medical informatics, user experience design, and Android mobile app development. The experts were enrolled via requests on social networks (eg, Instagram, Facebook, and LinkedIn) sent from October 20 to 28, 2020; we tried to enroll as many experts as possible.

According to the literature, we required between 14 and 28 experts [26]. Ultimately, 14 people agreed to participate in the design phase. In this phase, we used the Balsamiq Mockups software (Balsamiq Studios, LLC) to draw the user experience flow, and Figma (Figma, Inc) to prototype the user experience. Once the experts accepted an alpha version of the app’s prototype, we evaluated it using the heuristic method, in order to determine those elements of the app that needed improvement before the development phase.

We asked 5 experts in the field of user experience design and development of mobile health (mHealth) apps for assistance. They ranked the prototype using Jacob Nielsen’s 10 general principles of severity for evaluating interactive design, which use a 5-point scale [27,28]: 0 (“I do not agree that this is a usability issue”), 1 (“Not to be fixed unless extra time is available for the project”), 2 (“This should be fixed, but at the lowest priority”), 3 (“Must be fixed, so it should be given high priority”), and 4 (“This must be fixed before the product can be released”).

### Development Phase

The results of the analysis and design phases provided the knowledge to be used in the development phase, in which we developed a database using My Structured Query Language (MySQL) and a mobile app, which we called “MyShield,” using Android Studio. The development phase took place from November 1 to 8, 2020.

### Implementation Phase

During this stage, we recruited ordinary people (ie, nonexperts) for our study through purposive sampling. Interested candidates contacted us through online registration after seeing the recruitment posts on social networks (including Instagram, Facebook, and LinkedIn). Twenty-seven participants registered; this was within the intended range of 25 to 30 subjects, as used in other, similar studies [26,29]. The participants were aged 18 to 50 years and were required to own a smartphone running Android (minimum version 4.1).

We used an online workshop to introduce the research team and project aims. During the workshop, we explained the ADDIE design process to the participants and their involvement. As a pretest, conducted before using the app, the participants were required to complete a self-care inventory (SCI) questionnaire. This questionnaire includes 30 items and 6 dimensions [30]. The questions were answered using a 5-point scale (almost never, occasionally, half of the time, fairly often, and almost always). In this questionnaire, each dimension included 5 questions; the respondents scored each question using this scale. Then, we calculated each respondent’s score by adding the points from all 6 dimensions; the result shows the person’s level of self-care (Multimedia Appendix 1). The final score ranged from 30 to 150, with the following interpretations [30]: 120 or above, the person has personal well-being and serenity; 91 to 119, the person has some control of a good system of self-care; 50 to 90, the person is struggling and could use some assistance in developing a stronger self-care system; and under 50, the person experiences some serious difficulties in the area of self-care.

Once the participants completed the questionnaires, they started using the MyShield app, from November 10 to December 25, 2020 (45 days). During these 45 days, we asked the participants to use the app every day for as long as they wanted, but for at least 20 minutes per day; the time of day did not matter. The app contains an initial guide (presented as a showcase); we also created a WhatsApp group where the participants could ask us any questions related to the study and the app. The initial guide feature displayed the guidelines on how to work with the app after the user’s first encounter with it. The app also contained an “About Us” section, where users could find information about the research, the app design team, and how to contact the team. The app allowed users to update to a newer version in the case of any resolved technical issues.

### Evaluation Phase

After the implementation phase ended on December 26, 2020, we used the standard standalone MAUQ (Mobile Health App Usability Questionnaire) to evaluate the usability of the app. This questionnaire has 3 factors: ease of use (5 items), interface and satisfaction (7 items), and usefulness (6 items). It is based on a 7-item Likert scale ranging from 1 (strongly disagree) to 7 (strongly agree) [31]. In the end, we converted each factor into a percentage between 0 and 100 based on each item’s mean. We reused the self-care inventory questionnaire [30] in a posttest to analyze how the app influenced the participants’ self-care skills; we did so by comparing these results with the pretest conducted during the implementation phase. Both questionnaires were designed with Google Forms, whose web addresses were sent to the WhatsApp group. The data were analyzed using SPSS software version 22 (SPSS Inc).

### Ethics Approval

The participants in the pretest and posttest surveys provided informed consent. Tarbiat Modares University Ethics Committee (IR. MODARES. REC.1399.142) approved the entire study protocol.

## Results

### Design and Evaluation

In the analysis phase, meetings with the experts helped us find the optimal content of the MyShield app, including the main menu. The participants agreed that MyShield should cover the following 5 topics: self-motivation, daily life management, personal hygiene, healthy eating, and exercise.

We determined that self-motivation content should teach the users how to think positively, motivate themselves, and utilize their intrinsic motivations. In matters related to daily life management, the app should include strategies on how to manage stress, fear, and anger. Education is also essential in other areas, such as managing life during personal quarantine and social activities. The personal hygiene aspect should deal with education related to masking and observing social distancing, washing hands, and use of disinfectants. Healthy eating education should include information on useful supplements, beverages, fatty foods, proteins, dairy products, nutrition programs, and controlling or avoiding smoking. Exercise education should cover such topics as exercising at home, conditions for exercising outdoors, professional exercise, the intensity of exercise, and exercise in self-quarantine.

**Figure 2:**
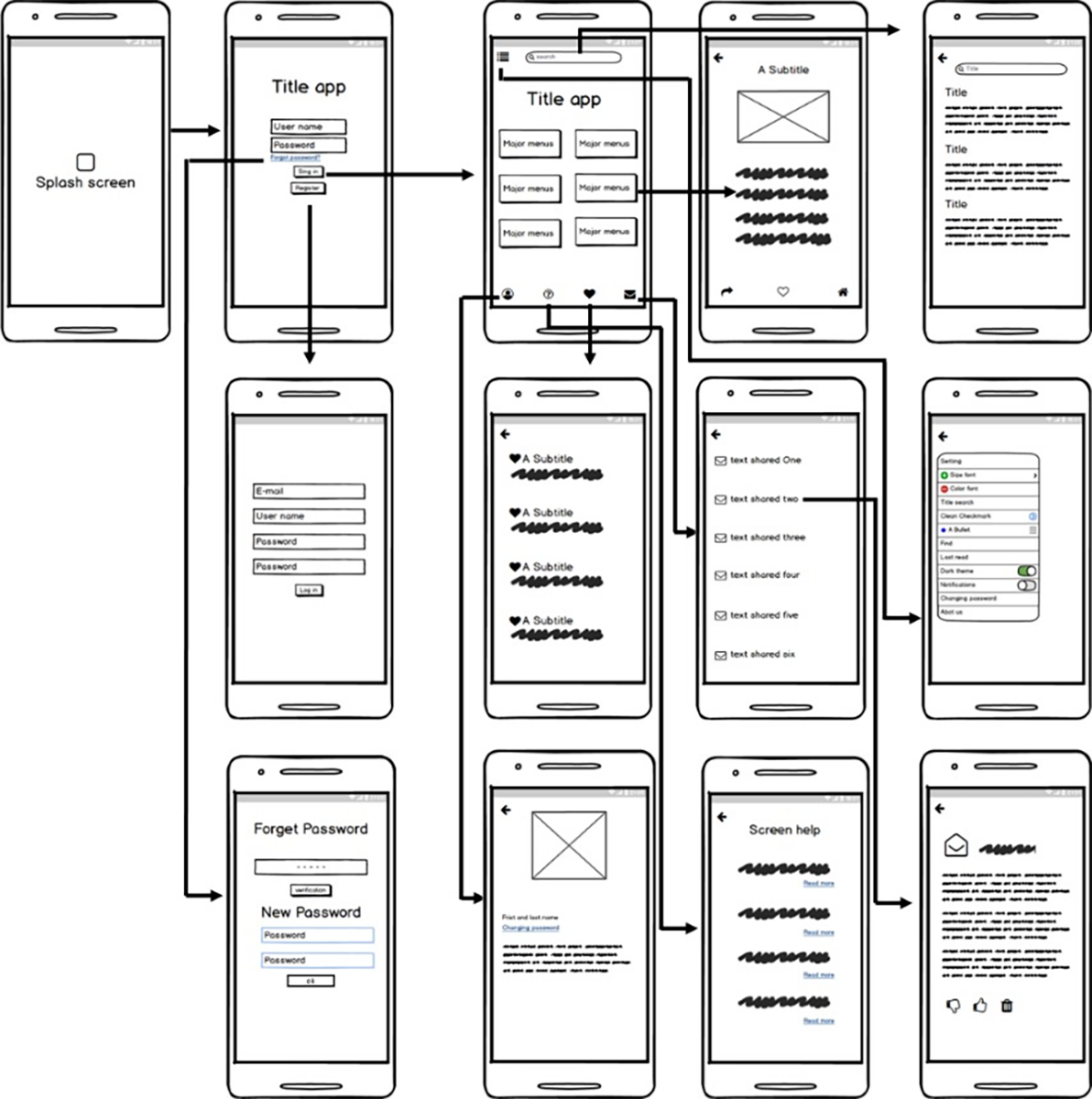
User experience flow of the MyShield mobile app.

Online workshops conducted with the 14 experts were utilized to determine the practical features; moreover, the user experience decided during the design phase helped us to determine the practical design of MyShield, as follows. The app starts with an initial opening screen. Next, the registration page appears. Here, the user must provide a few pieces of personal information (email, user name, and password). An introduction slide appears on the initial launch of the app. Three slides appear to be ideal for introducing the app. We found that in order to familiarize the user with the app, the showcase should appear after the introduction slide. After this, the user sees the main menu. It has two action bars: one, at the top of the page, displays the search feature of the entire app; while the other, at the bottom of the page, contains buttons, messages, help, settings, and profiles. On the left-hand side, there is a hamburger button (3 horizontal lines) which, when tapped, leads to the app’s settings. The settings include fonts, title searches, option selection and clearing, find, last read, light or dark theme, notification, changing passwords, and an “About Us” option. The content had a share button, which should be checked as marked. In addition, the users had an option of liking or disliking the content. Figure 2 shows the user experience flow. For example, tapping the “sign in” button opens the Main Page screen. Thus, this figure is both a user experience flow and a wireframe, which is an illustrated guide that shows the schematic and general framework of the MyShield app.

During the heuristic evaluation of the app’s prototype, these features each earned 0 to 2 points, meaning that the prototype received a rejection or an acceptable score. Table 1 presents the results: ΣA represents the total number of points for each item from Jacob Nielsen’s 10 general principles; ΣB is the same as ΣA with duplicated points eliminated. The total for ΣA was 45 and the total for ΣB was 23 (Table 1).

**Figure 3:**
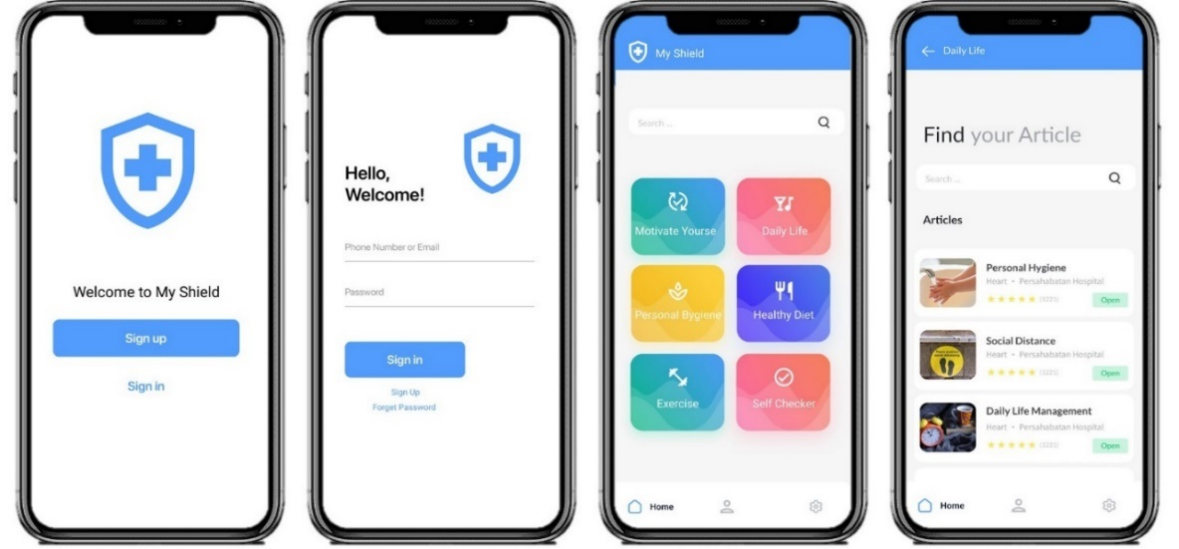
Examples of pages from the MyShield self-care mobile app.

In the development phase, functional requirements, user interface screens, and software database designs were created. The interface design was a process that involved installing specialized software on smartphones and connecting them to the internet, so that users could register their accounts in the app. Thus, the users were registered on the server and their registration IDs were stored on the app server. MySQL was used to design and develop the database, and Android Studio was used to develop the app. Eventually, after thorough consultation with infectious disease specialists and experienced nurses, a beta version of MyShield was developed and released (Figure 3).

**Table 1:** MyShield’s prototype scoring according to a heuristic evaluation based on Jacob Nielsen’s 10 general principles.

Heuristic principle	ΣA^a^	ΣB^b^	Expert identification number				
			1	2	3	4	5
Visibility of system status	4	3	0	1	2	1	0
Match between system and the real world	2	2	0	0	0	0	2
User control and freedom	4	3	1	1	0	2	0
Consistency and standards	2	1	0	1	0	0	1
Error prevention	6	3	2	2	1	1	0
Recognition rather than recall	3	1	0	0	1	1	1
Flexibility and efficiency of use	9	3	2	2	2	2	1
Aesthetic and minimalist design	6	3	1	0	1	2	2
Error identification, diagnosis, and recovery	3	1	0	1	0	1	1
Help and documentation	6	3	2	2	1	1	0
Total	45	23	8	10	8	11	8

^a^ΣA: total number of points.

^b^ΣB: number of points after removing duplicates.

### MyShield Usability and Satisfaction

Table 2 shows that the mean age of the 27 participants was 29.8 years; 12 (44%) participants were aged between 29 and 38 years. Fifteen participants (56%) were women; 19 (70%) had a bachelor’s degree or higher; and 12 (44%) had used a smartphone for at least 5 years.

The participants completed the MAUQ questionnaire designed for standalone mHealth apps. Overall, all items received a score of 3.5 or higher (Table 3). The “interface and satisfaction” component was rated the strongest with 95.9%, followed by “ease of use” (89.5%) and “usefulness” (86.7%).

**Table 2:** Demographic characteristics of the participants (N=27).

Characteristics, n (%)			Values
**Age (years)**			
	18-28	7 (26)	
	29-38	12 (44)	
	39-50	8 (30)	
**Gender**			
	Male	12 (44)	
	Female	15 (56)	
**Education**			
	High school diploma	3 (11)	
	Some college credits, no degree	5 (19)	
	Bachelor’s degree	10 (37)	
	Master’s degree	5 (19)	
	Professional degree	3 (11)	
	Doctoral degree	1 (4)	
**Duration of smartphone use (years)**			
	1-2	5 (19)	
	3-5	10 (37)	
	5 or more	12 (44)	

**Table 3:** The mean and SD of the results obtained from the MyShield usability app according to the Mobile Health App Usability Questionnaire. These statements can receive responses ranging from 1 (strongly disagree) to 7 (strongly agree).

Items, mean (SD) score			Values
**Ease of use**			
	1. The app was easy to use.	6.54 (0.499)	
	2. It was easy for me to learn to use the app.	6.33 (0.471)	
	3. The interface of the app allowed me to use all the functions (such as entering information, responding to reminders, viewing information) offered by the app.	6.29 (0.568)	
	4. The navigation was consistent when moving between screens.	6.15 (0.422)	
	5. Whenever I made a mistake using the app, I could recover easily and quickly.	6.00 (0.756)	
**Interface and satisfaction**			
	1. I like the interface of the app.	6.95 (0.213)	
	2. The information in the app was well organized, so I could easily find the information I needed.	6.93 (0.258)	
	3. The app adequately acknowledged and provided information to let me know the progress of my action.	6.89 (0.348)	
	4. Overall, I am satisfied with this app.	6.83 (0.373)	
	5. The amount of time involved in using this app has been fitting for me.	6.69 (0.462)	
	6. I would use this app again.	6.49 (0.523)	
	7. I feel comfortable using this app in social settings.	6.18 (0.383)	
**Usefulness**			
	1. The app improved my access to health care services.	6.51 (0.5)	
	2. The app would be useful for my health and well-being.	6.44 (0.585)	
	3. The app helped me manage my health effectively.	6.20 (0.72)	
	4. I could use the app even when the internet connection was poor or not available.	5.88 (0.391)	
	5. This app has all the functions and capabilities I expected it to have.	5.75 (0.652)	
	6. This mHealth app provided an acceptable way to receive health care services, such as accessing educational materials, tracking my own activities, and performing self-assessment.	5.63 (0.483)	

### Impact Rate of MyShield: Pretest and Posttest

**Figure 4:**
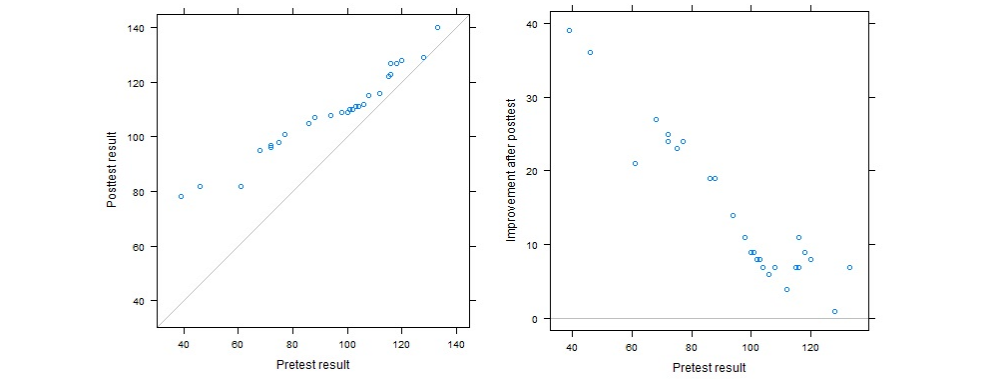
Pre- and posttest participants’ self-care assessment. The left panel shows posttest results against pretest results. Points above the gray line show an improvement in the posttest. The right panel (a modification of the modified Tukey mean-difference plot, presented by Kozak and Wnuk [32]) shows the improvement after the posttest against the pretest result.

In the pretest, the participants completed an SCI questionnaire before using the app: 2 respondents scored below 50, 8 scored 50 to 90, 14 scored 91 to 119, and 3 scored above 120 (Figure 4). In the posttest, the participants’ self-care scores increased notably at low levels, but those at high self-care levels increased only a little: 2 respondents who had a score below 50 in the pretest stage reached scores above 50 (even reaching close to 90) (Figure 4).

**Figure 5:**
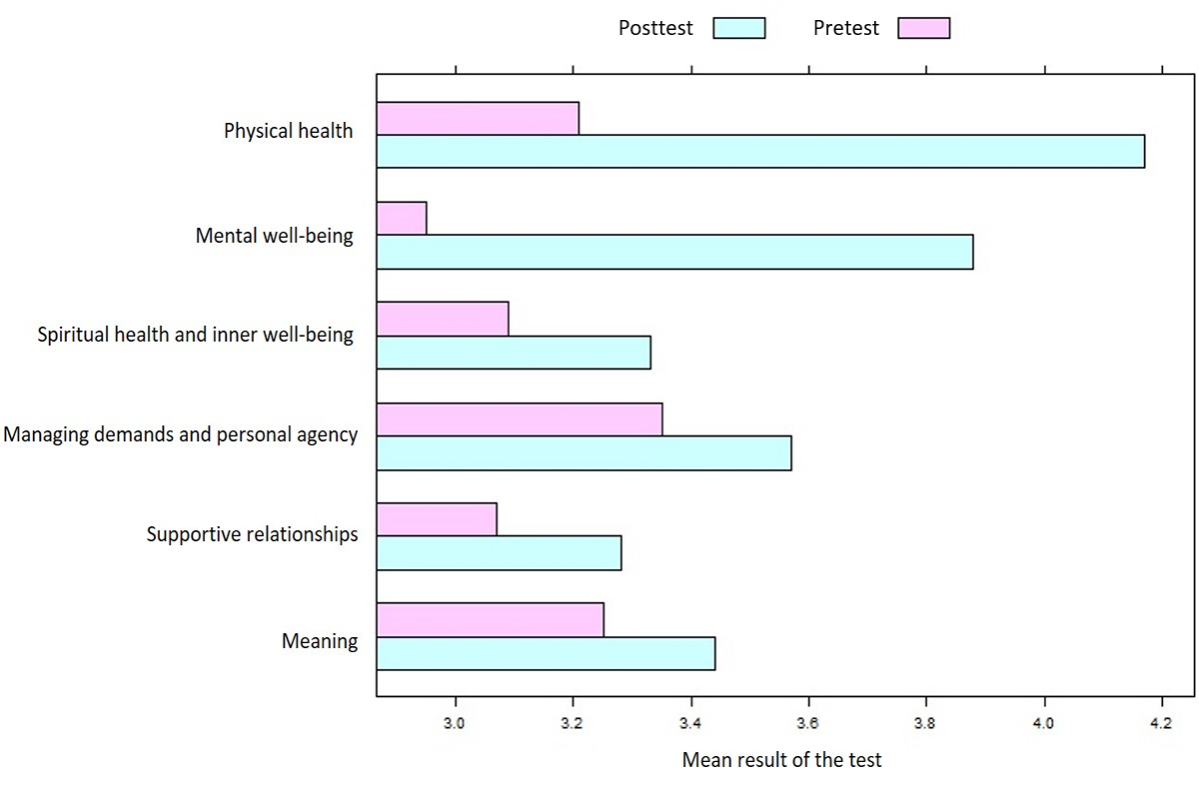
Comparison of mean scores for the self-care dimensions of the MyShield app. The graph is ordered by the dimensions’ improvement among the users.

After the users used MyShield, we examined their average scores (0-5) for the self-care dimensions in the pretest and posttest to determine which dimensions had improved and which had decreased. The mean mental well-being score increased from 2.95 in the pretest to 3.88 in the posttest. For the physical health dimension, the mean score increased from 3.21 to 4.17. The increases for the other dimensions were visibly smaller (Figure 5).

## Discussion

### Principal Findings

This study aimed to develop a self-care app in order to help users improve their skills to prevent COVID-19 infection. To this end, we have developed, with the help of various field experts, a mobile app called MyShield; it is designed to work mainly in underdeveloped countries, as people in such countries are often left alone to struggle with their fear of COVID-19.

We designed MyShield using the ADDIE educational model. This is a novel, interdisciplinary approach to app design. MyShield is a mobile app that, in addition to being a potential public health resource, is also an educational entity built to teach people self-care skills and health knowledge that is effective well beyond the use of the app. MyShield utilizes the ADDIE educational model to enable users to teach themselves these self-care skills, making the app a channel of knowledge the user can learn from. A possible effect that the app attempts to achieve is an increased chance for users to protect themselves from COVID-19. While MyShield is just a tool to initiate this learning, it is the source of the positive effects we have just discussed. We hope this plan will come to fruition, and that MyShield will encourage people from underdeveloped countries to believe in their own skills and learn or improve their self-care skills to reduce health risks.

As a standalone app, MyShield includes 6 major menus and various subcategories directly related to self-care training. In order to see how MyShield helped the participants, we conducted pretests before the initial use of the app. After participants used MyShield for 45 days, we conducted posttests to see if using the app helped them enhance their self-care skills. Both tests used a self-care inventory questionnaire to assess the participants’ self-care levels. All participants showed improvement; those who had shown poor self-care skills in the pretest improved much more than those with higher self-care awareness (Figure 5).

### Comparison With Prior Work

In the COVID-19 era, mobile apps are particularly useful when their design does not limit their use to a particular group or particular members of society, but rather allows them to be used by all society’s members, including young people, older adults, and people with physical disabilities. This issue is so crucial that it is emphasized as one of social justice and human rights [33,34]. To reach this goal, however, mobile apps need to be designed accordingly. In view of the user experience and user interface of MyShield, we tried to move the app user interface toward better usability and accessibility for various people, manipulating such features as font size and color, brightness, and dark and light themes [35]. We also tried to facilitate using the app so that the user did not need to perform complex functions. This led to an app design that did not require any special skills, such as working with Bluetooth or phone settings, and in which all functions were easily understood. We recognize one possible practical limitation of this study, which was the small number of participants, particularly older ones [36]. This suggests further work to be done in this area.

Islam et al [37] examined many mobile apps designed specifically for COVID-19. They classified the apps into various categories, such as remote assistance, patient monitoring, current status, COVID-19 prevention, COVID-19 control, communication support, and treatment services. None of these apps, however, directly emphasized training in self-care skills [37]. MyShield addresses this gap; it does so by directly focusing on the training of self-care skills. Thus, MyShield can be particularly useful in lower-income and underdeveloped countries, since studies have shown their citizens do not pay as much attention to training self-care skills [15]. This may result from many social and economic factors, even though this is perhaps the least expensive way to improve a society’s condition, particularly in the case of COVID-19 [14,18]. The impact ratings of the MyShield app indicate that it can improve people’s self-care skills by providing credible educational materials and enhancing citizens’ motivation to maintain their physical health and personal hygiene during the COVID-19 era. According to our research, MyShield’s ease of use rating reached 89.5%, a value suggesting that the app is easy to use. This includes easy learning, proper organization of information, and convenient access to information, features that many studies have emphasized are of crucial importance. Indeed, as other researchers have shown, ease of use strongly affects users’ perception of mHealth services [38-40]. Various studies have found that important items for assessing the quality of mHealth apps include information management, navigation consistency, guidelines, user interface design, error prevention, user control, freedom, performance speed, medical features, and content validity [41-44].

MyShield’s strength is in its interface, which had a satisfaction rating of 95.9%. According to the participants, the app organized information well, displayed progress, provided health information in a comprehensive manner, and let its users perform all its functions in an appropriate way. Access to health care services and educational materials is a unique feature of health apps [18,45-47]. MyShield was also rated well in these areas by the participants, who reported that they had no problems with accessing educational materials, tracking their own activities, and performing self-assessment.

The MAUQ results showed that the app met the health and care needs of individuals and helped them to improve their self-care skills by providing them with relevant information. We consider that such a systematic approach, taking into account people’s needs and limitations, reaches far beyond the confines of many contributions of COVID-19 self-care apps.

### Limitations

The study had a small number of participants. The number, however, was large enough to represent the app’s user groups in aspects such as education, gender, and age. However, we could have specifically included more older participants, most of whom are unfamiliar with health care apps on smartphones. This would have required a much larger study group. Our study designed the app specifically to address the needs of underdeveloped countries, but we conducted our survey in Iran, a more developed country than the target audience of this app. However, most participants—selected through purposive sampling—had little knowledge about self-care, so they can be considered a fair representation of people from underdeveloped countries.

Another limitation was the length of time (45 days) during which the participants had the opportunity to use MyShield. Budget limitations prevented us from extending the study beyond 45 days. Perhaps if the participants used the MyShield app for a longer period of time, the self-care inventory would have improved. In addition, the app was available for Android only, automatically excluding all iOS (ie, Apple) users. A future study may address these issues.

### Conclusion

This study demonstrates that using a standalone mobile app based on a standard educational model can improve people’s self-care skills. Improving self-care skills in underdeveloped countries with mobile health apps is a rather simple, inexpensive, and effective solution. It does, however, require a way to reach people and to convince them to use such an app. We consider that the design and development of MyShield can be used as a practical and realistic model for the development of mHealth apps. According to our findings, standard educational models combined with existing designs (eg, user-centered or interactive designs) for mobile health apps can meet most requirements of mobile educational apps.

As our research shows, MyShield can help people from both underdeveloped and developing countries, where the fight against COVID-19 is particularly difficult due to economics. We developed MyShield using a special educational design method to enable people to better learn to improve their self-care skills, thus helping to better protect themselves against COVID-19.

## Appendix

Multimedia Appendix 1 Self-care inventory.

## Abbreviations

ADDIE: analysis, design, development, implementation, and evaluation
MAUQ: Mobile Health App Usability Questionnaire
mHealth: mobile health
MySQL: My Structured Query Language
SCI: self-care inventory
