# Supplementary material for: Development of a Mobile App for Self-Care Against COVID-19 Using the Analysis, Design, Development, Implementation, and Evaluation (ADDIE) Model: Methodological Study
Source: JMIR Form Res. 2022 Sep 13;6(9):e39718. doi: 10.2196/39718 (PMC9472509; doi:10.2196/39718)
Supplement: Multimedia Appendix 1 [file formative_v6i9e39718_app1.docx]

**Self-Care Inventory**

**How well are you taking care of yourself?**

Change is created out of “optimal” anxiety. The purpose of this simple self-care inventory is to do just that — stimulate anxiety, but without immobilizing you. This is not an inventory that assesses every area of your life or who you are as a person. It is a simple inventory that will help you identify your current level of self-care in six key areas. It is meant to help you see any disparity between where you are and where you want to be in relation to self-care. It will help you focus on your needs and identify areas where change is called for.

The state of your self-care system varies at different times in your life. Sometimes you need to get lost in the forest in order to find a new path. Here are a few pointers to help you get the most from the inventory.
• Being honest with yourself is not easy, but to make changes in your life you need a clear inventory of where you are today. In some respects, this inventory will test how rigorously honest you are with yourself. We all lie to ourselves in one way or another as a way to survive. The more rigorously self-honest you are in answering these questions, the more you will gain from the experience. You don’t need to “look good” or impress anyone. There is no requirement to share your scores with anyone. It’s for you only.

• You may find yourself in conflict or getting anxious as you answer the questions. Remember that anxiety drives the engine of change. Respect the anxiety, and know there are no “right” answers or perfect scores (if you score high in every category maybe it means you need to lighten up and be less obsessive compulsive!) Hopefully the anxiety will inspire you to put yourself in the driver’s seat of change.

• Many people say they would answer many of the questions differently, depending on whether it is in relation to their work or in their personal life. If you feel there would be a big difference, you may want to take the test twice — once in the context of your work and once in the context of your personal life. This is okay, but if you see a large disparity between these two, there is an opportunity to reflect on what that means.

• Some of these questions will bring confusion and anxiety. Just as there are no perfect answers, this is not a perfect test. Even if it’s not clear, do the best you can.

• As you go through this inventory, you may see many areas in your current self-care system that could use a change. Rather than overhauling too much right now, focus on one or two areas that could use work in the next six to twelve months. Remember, it is better to be successful at one small change, than failing at trying to overhaul too many things!

• If you don't know the answers to any of the questions below, count your score a zero on that point.

| Almost Never | Occasionally | Half of the Time | Fairly Often | Almost Always |
| --- | --- | --- | --- | --- |
| 1 | 2 | 3 | 4 | 5 |

**Physical health**

This is where you examine the physical aspects of your life —how you are nurturing your body and taking care of your health.

| 1. I get seven to eight hours sleep most nights. | 1 | 2 | 3 | 4 | 5 |
| --- | --- | --- | --- | --- | --- |
| 2. I consistently exercise at least three times a week, for at least 20 minutes. | 1 | 2 | 3 | 4 | 5 |
| 3. I am a non-smoker. | 1 | 2 | 3 | 4 | 5 |
| 4. My body is a weight that is right for me. | 1 | 2 | 3 | 4 | 5 |
| 5. I have a healthy, well balanced diet. | 1 | 2 | 3 | 4 | 5 |

**TOTAL:**

**Mental wellbeing**

This area examines how your mind is supporting your self-care system, and some of the choices you make for having a strong mental attitude and firm mind in your life and work.

| 1. I am optimistic about my life and my future. | 1 | 2 | 3 | 4 | 5 |
| --- | --- | --- | --- | --- | --- |
| 2. Joy and gratitude come easily to me, and I do things that bring me joy. | 1 | 2 | 3 | 4 | 5 |
| 3. I am free of worry about people, places, and things that I have no control over. | 1 | 2 | 3 | 4 | 5 |
| 4. I am able to move from self criticism to self acceptance easily (i.e., I treat myself with the same respect and compassion I would a best friend.) | 1 | 2 | 3 | 4 | 5 |
| 5. I am able to deal with fear, guilt, and insecurity in a constructive way. | 1 | 2 | 3 | 4 | 5 |

**TOTAL:**

**Managing demands and personal agency**

This area deals with how you clarify the most important areas in your lifethat need attention, and how you manage the demands from others.

| 1. I am clear about the essential elements that matter most in my life. | 1 | 2 | 3 | 4 | 5 |
| --- | --- | --- | --- | --- | --- |
| 2. I attend to my core values on a regular basis. | 1 | 2 | 3 | 4 | 5 |
| 3. I have clear boundaries, when necessary, around the expectations of others. | 1 | 2 | 3 | 4 | 5 |
| 4. I am satisfied with the way I handle demands in my life. | 1 | 2 | 3 | 4 | 5 |
| 5. I have little in my life that needs cleaning up (household clutter, destructive | 1 | 2 | 3 | 4 | 5 |

**TOTAL:**

**Spiritual health and inner wellbeing**

Spiritual wellbeing is about tapping into a power beyond ourselves to give guidance, support, and clarity on the self-care journey. Spiritual health is about finding inner peace, independent of the roles, successes, and failures of life.

| 1. I get strength from my religious and/or spiritual beliefs, where I find peace in the midst of chaos. | 1 | 2 | 3 | 4 | 5 |
| --- | --- | --- | --- | --- | --- |
| 2. I take quiet time for myself during the day for strength and perspective from within. | 1 | 2 | 3 | 4 | 5 |
| 3. I have a level of acceptance of my imperfections (self-compassion) and have the strength to let go of my fear and uncertainty. | 1 | 2 | 3 | 4 | 5 |
| 4. Having the courage and faith to believe in what I cannot see gives me strength in my life. | 1 | 2 | 3 | 4 | 5 |
| 5. I have a community that supports my spiritual beliefs. | 1 | 2 | 3 | 4 | 5 |

**TOTAL:**

**Supportive relationships**

This area examines the quality of supportive relationships in your life, people that support you and hold you accountable to take care of yourself, and help guide you to your own truth.

| 1. I give and receive affection regularly. | 1 | 2 | 3 | 4 | 5 |
| --- | --- | --- | --- | --- | --- |
| 2. I have at least one relative within an hour’s drive on whom I can rely. | 1 | 2 | 3 | 4 | 5 |
| 3. I have a network of friends on whom I can — and do — depend. | 1 | 2 | 3 | 4 | 5 |
| 4. I have at least one friend that I confide in about personal matters, and I meet with them regularly. | 1 | 2 | 3 | 4 | 5 |
| 5. I am able to speak openly about my feelings when angry or worried. | 1 | 2 | 3 | 4 | 5 |

**TOTAL:**

**Meaning**

This area speaks to the level of satisfaction and fulfillment you are getting from your life and your work right now that makes the caring in your life meaningful.

| 1. I enjoy getting out of bed most days, and look forward to the day. | 1 | 2 | 3 | 4 | 5 |
| --- | --- | --- | --- | --- | --- |
| 2. I have a sense of purpose in my life. | 1 | 2 | 3 | 4 | 5 |
| 3. If I suddenly received an inheritance of $1 million, my life wouldn’t change much. | 1 | 2 | 3 | 4 | 5 |
| 4. I stand up for what I believe in. | 1 | 2 | 3 | 4 | 5 |
| 5. I express my unique talents, strengths, passions, and dreams on a daily basis. | 1 | 2 | 3 | 4 | 5 |

**TOTAL:**

**Grand Total (Add Up All Six Categories):**

Scores can range from 30 to 150

| 120+ | This score indicates personal wellbeing and serenity at this stage in your life. You have a good self-care system in place. You may identify an area that needs some “fine tuning,” but take time to appreciate your current life style, choices, and habits in the area of selfcare. You may also want to reflect on areas of self-care that need attention but did not get addressed in this inventory. Note: If you scored in this range, you may also be an obsessive compulsive person who could benefit by lightening up and perhaps putting less emphasis on discipline and structure. |
| --- | --- |
| 91-119 | You have some areas in your life with a good system of self-care. Some areas also need focus and new habits. Take a close look at one or two areas that need attention now, and focus on them. |
| 50-90 | This score would indicate that you are struggling and could use some assistance in developing a stronger self-care system. Look seriously at the area(s) in your life that need attention now. You may need to establish a structure to enhance your life through more discipline and consistency. |
| Below 50 | You are experiencing some serious difficulties in the area of self-care. Take time to determine if these difficulties are stemming from a current change in your life, and if so, ask how you can create some structure in your life to take better care of yourself in this challenging time. You will need to pay serious attention to some immediate action toward self-care in order to rekindle your personal vitality. Appreciate yourself for being so rigorously honest with yourself. This is the first step to growth |

Regardless of your score, here is a short list of actions to get you started on the next chapter of your self-care journey.

• Take time to do a more thoughtful inventory — paying particular attention to the scores that were lowest. Review any blocks that may be getting in your way of creating a self-care system that serves you best. Also, be sure to take time to acknowledge the strengths of your current self-care system. • Reflect on the score that you gave yourself in this inventory. Ask whether the assessment fits for you. Does this score reflect how you are currently taking care of yourself? Take your self-care seriously by taking a good honest look at yourself.

• Be sure to give yourself some credit for any areas in your life where you are attending to yourself.

• Pick one area in your life that needs some focused attention to self-care, and develop a plan for working in this area using the “Taking Action” process outlined below.

• Create a “sanctuary,” a place or time for you to get away from the demands of others to be still and listen to the voice within. This could be a physical space, a room in your house, and/or a time during the day or week that you can unplug from technology, distractions, and demands just to be with yourself.

• Connect with a support system to give you a fresh perspective, and help hold you accountable to work with the area that you are focusing on. This could be a trusted friend, therapist, coach, mentor, trainer at a local gym, massage therapist, a religious community, social club, or a support or recovery group.

• Search for a confidant, a person with whom you can share your innermost thoughts, feelings, dreams, and challenges.

• Sign up for a class that will help you, such as yoga, Tai Chi, mindfulness meditation, or relaxation.

• Remember, start small and be consistent. It’s better to walk even five minutes every day than an hour a month.

• Do a “clean up” in your life to make room for something new (e.g. de-clutter your house, get rid of anything that is not bringing you joy, let go of any relationships that are not life-giving, donate clothes that you haven’t worn in years to charity, have a garage sale, or clean out those old self-help books you no longer need).

• If self-compassion is an area you would like to develop (which is a particularly a big one for me), a useful tool can be found on Dr. Neff’s Web site: www.self-compassion.org. You may also find Brené Brown’s book, The Gifts of Imperfection, to be a valuable tool.

**Taking Action**

Take an honest look at each of the six following areas and do a quick analysis. Where are you and where do you want to be? Which areas show a gap between current reality and your vision? Start with one area that you are committed to work on. Which areas are going well? Acknowledge your progress in these areas. Note: seek out an “accountability partner,” a trusted friend or advisor who will support you and help keep you accountable.
